# Natural Products as Potential Resource Library for Control of Major Swine Enteric Viruses

**DOI:** 10.1155/tbed/4368881

**Published:** 2026-02-03

**Authors:** Jialu Zhang, Yuqian Liu, Shuying Ren, Zhouyuan Wang, Yunxia Li, Lianci Peng, Rendong Fang

**Affiliations:** ^1^ Joint International Research Laboratory of Animal Health and Animal Food Safety, College of Veterinary Medicine, Southwest University, Chongqing, 400715, China, southwest.edu; ^2^ Kunming Hemeihua Feed Limited Company, Kunming, 682100, China

**Keywords:** antiviral medicine, natural products, swine enteric viruses (SEVs), traditional Chinese medicine (TCM)

## Abstract

Major swine enteric viruses (SEVs), including porcine epidemic diarrhea virus (PEDV), porcine deltacoronavirus (PDCoV), transmissible gastroenteritis virus (TGEV), swine acute diarrhea syndrome coronavirus (SADS‐CoV), and porcine rotavirus (PoRV), cause severe gastrointestinal diseases in pigs, leading to huge economic losses to the swine industry around the world. In the absence of specific drugs and vaccines for controlling SEVs in the pig production, this review summarizes the inhibitory effects of natural products against these major porcine enteric viruses. Specifically, it focuses on recent studies regarding the anti‐SEVS activities of traditional Chinese medicine (TCM) compound formulas, herbal extracts, pharmaceutical monomers, and natural metabolites. The review elaborates on how these natural products exert antiviral activities against SEVs, highlighting their potential as alternative or complementary agents for controlling porcine enteric viral infections. Overall, this work provides a comprehensive overview of the research progress in natural products against porcine enteric viruses and demonstrates the new strategies for medicine discovery, which will be helpful for further development of effective antiviral strategies in the swine industry.

## 1. Introduction

**Figure 1:**
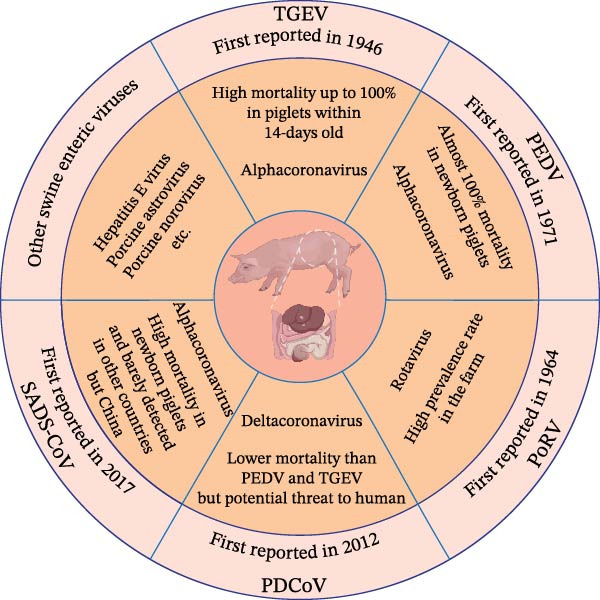
Prevalent characteristic of major SEVs in recent years.

The small intestine consists of abundant cells and microbes for nutrients absorption and immunity homeostasis. Enteric infections affect porcine growth performance and causes giant economic loss to pig industry in the world. Swine enteric viruses (SEVs) are highly contagious and characterized by similar symptoms such as weight loss, depression, diarrhea, vomiting, dehydration, and death within severe cases [1]. The gross dissection typically reveals hyperemia or hemorrhage in the intestine, which is filled with yellow fluid, besides, the pathological examination constantly shows necrotic intestinal epithelial cells and atrophic intestinal villi [2]. Diseases caused by enteric viruses differ from the fulminant infectious diseases in pigs, which induce acute morbidity and rapid death in pigs such as rabies virus [3], Japanese encephalitis virus [4], and foot‐and‐mouth disease virus [5]. SEVs infections result in continuous symptoms for several days, of which the threat might be neglected but great potential risk to public health. So far, five major enteric viruses in pigs have been identified, containing four coronaviruses, transmissible gastroenteritis virus (TGEV), porcine epidemic diarrhea virus (PEDV), porcine deltacoronavirus (PDCoV), and swine acute diarrhea syndrome coronavirus (SADS‐CoV), and one rotavirus, porcine rotavirus (PoRV) [6, 7]. PEDV has been prevalent in the world for nearly 50 years and caused economic losses of millions of dollars every year. Of note, PEDV‐induced diarrhea causes almost 100% mortality in newborn piglets and has become a major swine disease in China and United States (US) [8]. Since the first outbreak of PDCoV in US in 2014, it has spread throughout the country rapidly, after that, PDCoV has been introduced into other countries such as South Korea, Thailand, and China [9]. PDCoV strains identified in South Korea and Thailand showed high sequence identity to the strains prevalent in US, besides, strains detected in mainland China has been confirmed to share high nucleotide homology with those identified in Hong Kong, China in 2012 [10, 11]. A retrospective study verified the presence of PDCoV‐positive porcine serum as early as 2010, whereas it was neglected because of lower prevalence rate and lethality rate than PEDV [12]. A study reported that PDCoV was detected in the blood of some Haitian children, indicating threat to public health safety [13]. Since 2017, SADS‐CoV was firstly discovered in Guangdong province, China and caused severe watery diarrhea and death in piglets [14]. Subsequently, SADS‐CoV was detected in Fujian Province, China in 2018 and re‐emerged in Guangdong Province, China in 2019 [15, 16]. The sequences of all SADS‐CoV strains share a high identity of 99.5% and it has been rarely reported in other countries in the world so far [17]. Similar to PDCoV, TGEV was firstly detected in the US in 1946 and then spread throughout the world, while its infection causes high mortality up to 100% in piglets within 14‐days old, same as PEDV, but lower prevalence rate [18]. PoRV was firstly isolated from porcine feces in 1974 and has high prevalence rate in the farm and positive antibody can be easily detected in the adult pigs, which indicates the extensive existence of PoRV for long time [19]. Besides, it was suggested that infected animals possess potential abilities to transmit rotavirus to human [20] (Figure 1).

As is known, developing specific and effective vaccines is important for the control of viruses, whereas efficient commercial vaccines are rare in the prevention of SEVs [21]. For newborn piglets, time is too short to develop intestinal immunity to encounter viral invasion, thus immunization of sows in advance could improve the passive immunity of piglets by supplying colostrum or milk [22–24]. In general, several types of vaccines were developed, including inactivated vaccine [25–28], live‐attenuated vaccine (such as FJzz1 for PEDV [29], GDS04 for SADS‐CoV [30], candidate rCHN‐HN‐1601 for PDCoV [31], and a trivalent vaccine for PoRV [32]), and subunit vaccine (mainly designed based on S protein for prevention of coronaviruses [33–35] and recombinant protein for PoRV vaccination [36]). Besides, novel mRNA vaccine also was designed and exhibited protective effect on piglets challenged by PEDV [37]. However, most of the vaccines were only developed in the laboratory and lack evaluation via virulent virus challenge, their effectiveness on farms during viral outbreaks remains to be verified. Besides, chemical drugs are limited to the application in pig industry due to their tendency to develop drug resistance and residual risk. It is urgent and necessary to explore low‐toxicity and valid medicines as alternative scheme for controlling SEVs infection.

Natural products have attracted the attention of researchers due to their various advantages, including low toxicity, multiple bioactivities, and minimal side effects. Furthermore, their wide availability contributes to controllable development costs [38]. Natural products have been applied and explored to treat various diseases during the lengthy existence of China, and the efficacy of natural products has been confirmed by numerous clinical cases [39]. After the artemisinin was found by Youyou Tu [40], the number of studies on traditional medicine has increased in a fabulous tendency. During the outbreak of Covid‐19 from 2019 to 2023, traditional Chinese medicine (TCM) also played a positive role in the control of SARS‐CoV‐2 [41]. In addition to traditional medicine, herbal compounds, plant‐derived constituents, and functional metabolites also belong to natural products, which exhibited great potential of antiviral capacity.

Enteric viruses exhibit high stability under environmental changes and harsh conditions, especially the porcine enteric viruses, which can cause potential outbreak by contaminating soil, water, and food [42]. Hence, this review aims to summarize the mechanism and application of natural products in restricting major enteric viruses in pigs. Additionally, this review offers further perspectives and potential strategies for effective control of enteric viruses.

## 2. Restriction of Major SEVs by TCM and Herbal Extracts

TCM has a long history of treating various diseases in China, with its origins dating back to approximately 3000 BC [43]. Since the accomplishment of Shen Nong Ben Cao Jing in Han Dynasty, the administration of TCM became extensive and prevalent [44]. Actually, in the past history of China, TCM was frequently used to control infectious diseases with complete theoretical system containing prevention of “Wen Bing” [45]. An ancient TCM formula described a method for treating malaria: immerse a handful of Qinghao (*Artemisia annua L*.) in 2 L of water, wring out the juice, and drink it entirely. Inspired by this method, Professor Youyou Tu isolated artemisinin from Qing Hao and rescued millions of lives in the world [40]. TCM was also proved effective in the control of terrible viral pandemics, which greatly lower the mortality caused by SARS in China [46]. In the past few years, TCM, such as Lianhua Qingwen capsules, Xuanfeibaidu granules, Xuebijing, were adopted and popularized by Chinese government to fight with Covid‐19 and achieved effective work [47]. Like artemisinin, the active ingredients of these recipes, such as glycyrrhizin, chlorogenic acid, and kaempferol, were clarified to exert antiviral roles by binding viral proteins and regulating various host signaling pathways. During the development of pig industry, accurate and effective medicines available for treating major SEVs are deficient, which could benefit from the exploration of TCM and ingredients.

### 2.1. TCM Recipes Effective in Treating Major SEVs Infection

The TCM recipes including two main formulations: decoction and modern granular formulation. Decoction is the most traditional production method for administration of herbal medicines, which contains a series of boiling and filtering and exhibits great absorption efficiency. The preparation of granular formulation is composed of a single herb or several herbs and is combined with the decoction. Granules has great convenience to address and store for months and even years, in addition, granules are easy to take for patients [48]. The major difference between decoction and granules is the main active ingredients after processing, thus the final chemical components determine the therapeutic effect of TCM formulations.

Fuzheng Jiedu decoction (FZJDD), a TCM recipe consisting of Danfupian, dried ginger, roasted licorice, honeysuckle, saponin thorn, hairy peach, patchouli, and tangerine peel, was effective in combating COVID‐19, which was achieved by blocking the binding of SARS‐CoV‐2 S protein to ACE2 receptor, inhibiting the activity of SARS‐CoV‐2 3CL^pro^. Suppressing inflammatory response via regulating TNF and MAPK signaling pathways also contributed to the antiviral capacity of FZJDD [49]. The anti‐PEDV capacity of FZJDD was tested in Vero E6 cells, data showed that the EC_50_ of FZJDD against PEDV is 0.16 mg/mL, besides, it also inhibited SADS‐CoV infection in Huh7 cells with the EC_50_ of 0.22 mg/mL. FZJDD showed antiviral effects on the whole infectious cycles of PEDV and SADS‐CoV [49] (Table 1). Further investigation of compounds from FZJDD identified four agents (kaempferol‐7‐O‐glucoside, icaritin, kaempferol, and octyl gallate) effective in PEDV inhibition at a concentration of 20 μM and four ingredients (nobiletin, arctigenin, caffeic acid, and echinatin) effective in inhibiting SADS‐CoV infection with inhibitory rate more than 90% at a concentration of 10 μM. Among them, kaempferol‐7‐O‐glucoside, icaritin, kaempferol, and octyl gallate targeted the whole life cycle of PEDV, whereas obiletin, caffeic acid, and echinatin exerted antiviral abilities during the viral entry and post‐entry periods [49].

**Table 1:** Effective recipes or compounds from TCM for SEVs inhibition.

Recipes (or compounds)	Category	Models	Targeting stages	Viruses	References
Fuzheng Jiedu decoction	Recipes	In vitro	Attachment, entry, replication	PEDV, SADS‐CoV	Liu et al. [50]
Pulsatilla Powder	Recipes	In vivo	—	PEDV	Chang et al. [51]
Lizhong decoction	Recipes	In vitro and in vivo	Replication	PEDV	Chen et al. [35]
*Hypericum japonicum* extract	Water extract	In vitro and in vivo	Replication	PEDV	Rao et al. [52]
*Epimedium koreanum* Nakai extract	Water extract	In vitro and in vivo	—	PEDV, TGEV	Cho et al. [53]
*Lonicera japonica* extract	Water extract	In vitro and in vivo	—	PEDV	Cho et al. [53]
Aloe extract	Water extract	In vitro and in vivo	Replication	PEDV	Xu et al. [54]
	Ethanol extract	In vitro	Replication	SADS‐CoV	Zheng et al. [55]
Chestnut inner shell extract	Ethanol extract	In vitro	—	PEDV	Kim et al. [56]
*Glycyrrhiza uralensis* Fisch.	Ethanol extract	In vitro and in vivo	Attachment, entry, and replication	PEDV	Bai et al. [57]
*Glycyrrhiza uralensis* Fisch.	Ethanol extract	In vitro and in vivo	—	PoRV	Alfajaro et al. [58]
leaf extract of *Moringa oleifera* Lam.	Water extract	In vitro	Replication	PEDV	Cao et al. [59]
Glycyrrhizin	Triterpene saponin	In vitro	Entry and replication	PEDV	Huan et al. [60]
Cepharanthine	Bisbenzylisoquinoline alkaloids	In vitro and in vivo	Attachment, entry, and replication	PEDV and PDCoV	Dong et al. [61] Sun et al. [62]
Tetrandrine	Alkaloid	In vitro	—	PEDV and SADS‐CoV	Qian et al. [63] Leng et al. [64]
Fangchinoline	Alkaloid	In vitro	Entry and replication	PEDV	Zhang et al. [65]
Puerarin	Isoflavone	In vitro and in vivo	Replication	PEDV	Wu et al. [66]
Matrine	Alkaloid	In vitro	—	PEDV	Qiao et al. [20]
Dehydroevodiamine	Alkaloid	In vitro	Entry and replication	PEDV	Li et al. [67]
Curcumin	Polyphenol	In vitro	—	PDCoV	Wang et al. [68]
		In vivo	—	PEDV	Jiang et al. [69]
		In vitro	Attachment and replication	TGEV	Li et al. [70]
Myricetin	Flavonoid	In vitro	Replication	TGEV	Fan et al. [71]
Chrysin	Flavonoid	In vitro	Replication	PEDV	Gong et al. [72]
Naringenin	Flavonoid	In vitro	Replication	PEDV	
Polysaccharides of *Portulaca oleracea* L.	Polysaccharides	In vivo	—	PoRV	Li et al. [73]
Ergosterol peroxide	Sterols	In vitro and in vivo	Attachment and entry	PDCoV	Duan et al. [74]
		In vitro	Entry and replication	PEDV	Liu et al. [75]

Pulsatilla powder, composed of *Pulsatilla*, *Coptidis rhizome*, and *Cortex phellodendri chinensis*, was applied in the treatment of diarrhea [76]. Of interest, Pulsatilla powder decoction promoted growth performance and reduced diarrhea of piglets challenged by PEDV, meanwhile, the declined gene expression of PEDV M in the intestine indicated that PEDV replication was impeded. Application of the decoction repaired intestinal villi destroyed by PEDV and promoted the antioxidants level in intestines. Moreover, the product of Pulsatilla powder fermented by *Lactobacillus casei* showed greater effectiveness against PEDV [51]. According to TCM theory, PEDV‐induced diarrhea was diagnosed as damp heat diarrhea. Lizhong decoction (LZD), mainly comprised of *Panax ginseng* C. A. Mey., *Rhizoma zingiberis*, *Radix glycyrrhizae preparate* and *Atractylodes macrocephala koidz*, was applied in multiple gastrointestinal diseases, such as irritable bowel syndrome, ulcerative colitis, and infantile rotavirus diarrhea [77]. LZD suppressed the infection of PEDV in vitro by inhibiting the replication stages in a dose‐dependent manner. The application of LZD significantly alleviated the clinical symptoms (diarrhea and vomiting) of PEDV‐infected piglets and increased the survival rate, moreover, the viral loads in lung, kidney, liver, spleen, and jejunum were eliminated [78].

These ancient recipes showed great potential to treat virus‐induced diarrhea, while the therapeutic effect of these recipes on SEVs infection were investigated using PEDV model because of its high threat and mortality to piglets, whether these prescriptions are effective in resisting other SEVs should be further verified. Besides, the modern herbal medicine fermentation technology also showed great advances in raising the content of effective ingredients, which helps to raise the therapeutic effect and lower the cost.

### 2.2. Compounds From Single Herb Effective in Inhibiting SEVs Infection

In the modern TCM studies, the researchers focus on the pharmacological effect of single herbs (especially single ingredients) in the recipes in order to uncover the underlying antiviral mechanisms and accelerate the development of medicines.

#### 2.2.1. Mixture Extract From Single Herb

The most common methods for the extraction of TCM are water decoction and ethanol tincture, by which the herbs are simmered in water or soaked in high‐level alcohol, respectively. Besides, oil infusion is applied to acquire fat‐soluble ingredients.

The *Hypericum japonicum* was extracted with water and rotary vaporization. In an in vitro model, the extract showed direct virucidal ability and inhibitory effect on PEDV replication in vero cells and IPI‐FX cells, besides, the late stage of PEDV lifecycle was mainly targeted by the extract. Furthermore, the extract was administered preventatively before the infection by PEDV in a piglet model, which exhibited full protective effect on piglets and alleviated vomiting and diarrhea [52].

*Epimedium koreanum* Nakai and *Lonicera japonica* were extracted by water decoction method and recognized as possessing anti‐PEDV abilities with reduced cytopathic effects screened from 333 natural herbs. Meanwhile, the extract of *Epimedium koreanum* Nakai also inhibited infection of TGEV. The extract was fed with diet in PEDV‐infected piglet model, consequently there were no intestinal damage and viral particles were detected in the piglets at 24 hpi. With the experiment prolonged, the viral number was increased but still far below the PEDV‐infected piglets without extract fed [53].

*Aloe*, an herb broadly applied in skin repair [79], was shown anti‐PEDV capacity by using its aqueous extract. Despite its direct virucidal effect, the *Aloe* extract suppressed the PEDV replication in the late period of viral infection at concentration of 16 mg/mL in vitro. After that, *Aloe* extract was orally applied in the PEDV‐challenged piglets at a safe concentration of 100 mg/kg body weight tested by using BALB/c mice. *Aloe* extract protected piglets from PEDV‐induced diarrhea, vomiting and death, besides, histopathological injury and viral particles in intestine were significantly reduced. Of interest, slight diarrhea was found in *Aloe*‐treated piglets, which was attributed to the purgative ingredient emodin [54]. Another study focused on the anti‐SADS‐CoV activity of emodin from *Aloe*. The data showed that ethanol extract of *Aloe* suppressed the SADS‐CoV infection in vero and IPI‐FX cells. Meanwhile, emodin from the *Aloe* extract inhibited the replication stage of SADS‐CoV but had no effect on the viral infectivity. The expressions of cellular TLR3, IFN‐λ3, and ISG15 were upregulated, however, the antiviral effect of emodin in vivo was not verified [55].

Chestnut was applied to treat the diarrhea and anorexia according to the theory of TCM [80, 81]. Recently, the ethanol extract of chestnut inner shell (ECIS) exhibited natural antiviral activity against PEDV infection, especially the attachment and membrane fusion. Of note, the ECIS also showed its antiviral potential on other coronaviruses such as SARS‐CoV, SARS‐CoV‐2, and MERS‐CoV, evaluated by using pseudotyped viruses [56]. In addition, another herb, *Glycyrrhiza uralensis* Fisch. (GUE; also called licorice), broadly mixed with other herbs in the recipes to nourish the vitality and coordinate the drug properties [82]. The ethanol extract of GUE showed anti‐PEDV activity via inhibition of viral attachment, internalization, and replication in vitro, meanwhile, the in vivo administration also exerted great antiviral potential by alleviating pathological damage and declining the viral loads in the intestine [57]. In the diarrhea caused by PoRV, treating with GUE at concentration of 400 mg/mL also acquired therapeutic effects on intestinal injury and viral excretion [58]. The increased mRNA expressions of inflammatory cytokines such as IL8, IL10, and IFN‐β caused by PoRV infection were dramatically reversed by GUE application in a dose‐dependent manner. The aqueous leaf extract of *Moringa oleifera* Lam. (MOE) tree played roles in inhibition of PEDV replication. MOE showed antiviral activity in vero cells when co‐cultured with PEDV, the further data demonstrated viral replication inhibition by MOE. Mechanically, MOE exerted anti‐PEDV effect through relieving the oxidative stress and inflammatory cytokines production and promoting the antiapoptotic level of the cells [59].

The mixture extract shows great potential on the disease control in the animal production, whereas the medicinal efficacy waste of this method brings high cost and vague mechanisms.

#### 2.2.2. Effective Antiviral Chemical Component of Herb

The modern TCM research believes that main bioactive constitutions of herb are associated with its pharmacological effects. Combining chemical fingerprints with specific bioactivities is frequently used in the TCM analysis [83], and thus the quality control of some herbs was built, for example, chlorogenic acid is one of the main ingredients and is utilized as the quality control material of *Eucommia ulmoides* according to Chinese Pharmacopoeia. However, because of the complexity of TCM, the specific effective component from candidate herbs still needs related assays to be clarified.

Glycyrrhizin, a major component extracted from licorice root, was identified as potential antiviral candidate against PEDV. Similar to the effect of GUE, glycyrrhizin also impeded the entry and replication of PEDV. Moreover, glycyrrhizin functions as competitive inhibitor of HMGB1, which was utilized by PEDV through TLR4 and TAGE during the infection [60].

*Stephania japonica* was employed for heat‐clearing and detoxifying in the past years, in recent years, Bisbenzylisoquinoline alkaloids extracted from *Stephania japonica*, including cepharanthine (CEP) and tetrandrine (TED), showed antiviral capacities [84]. CEP had been applied to raise leukocyte counts of cancer patients after the chemotherapy for decades since its discovery. CEP was shown anti‐PEDV [61] and anti‐PDCoV [62] ability by blocking the whole viral life cycle. In a PEDV‐challenged piglet model, CEP treatment at a concentration of 11.1 mg/kg bw protected piglets from intestinal damage and diarrhea. TED, extracted from the root of *Stephania japonica*, exhibited anti‐PEDV and anti‐SADS‐CoV effects in vero cells [63, 64]. The extractive resource of fangchinoline (Fan) was similar to TED, which mainly targeted the replication stage of PEDV. Autophagy was involved in the PEDV infection, the late stage of which was blocked by Fan and the autophagosomes were accumulated consequently [85]. Besides, lysosomal pH was increased and proteinase activities were inhibited by Fan to refuse the entry of PEDV [86]. Considering that the roles of autophagy and lysosome in coronavirus life cycle, the infection of SADS‐CoV was also interfered by TED tested in a vero cell model [64].

Puerarin (PR) was extracted from the *Lobed Kudzuvine* root, a Chinese herb applied in treating cardiovascular diseases and infection [87, 88]. Similar to *Lobed Kudzuvine*, PR possesses multiple biological activities, including but not limited to anti‐inflammation, antitumor, blood pressure control, and antivirus [89]. The oral application of PR at a concentration of 0.5 mg/kg body weight significantly relieved the symptoms of 7‐day‐old piglets challenged by PEDV. In detail, the PR restored the growth performance of infected piglets and repaired the intestinal villi. The in vitro study using vero cells also indicated that PR inhibited PEDV replication and the cytokines expression induced by PEDV. Proteomics analysis of ileum demonstrated that PR application benefits the up‐regulation of interferon‐stimulated genes [66]. Matrine (MT), a natural alkaloid extracted from the root of sophora flavescens, has various pharmacological activities such as anti‐inflammation, antibacteria, antiparasite and antivirus [90]. The additional analysis suggested that the antiviral effect of MT is not affected by the time. Besides, the molecular docking indicated the docking site GLY434 of the PEDV spike protein with MT is conserved, which probably was targeted by MT. MAPK signaling pathway was activated by MT to accelerate the apoptosis of PEDV‐infected cells, resulting in the inhibition of PEDV infection. Meanwhile, the extract of sophora flavescens also showed moderate anti‐PEDV capacity [91]. Another alkaloid, dehydroevodiamine (DHED), is extracted from the fruit of *Evodiae Fructus* (also named as Wu‐Zhu‐Yu in TCM) [92]. DHED showed various pharmacological activities, including anticancer and anti‐inflammation [93]. Previous studies also discovered the protective effect of DHED on intestine and stomach [94–96], which suggested the possible function in recovering SEVs‐induced porcine diarrhea. An in vitro study showed that DHED application at the concentration of 6.25 μg/mL was effective in inhibiting PEDV activities and the inhibitory effect is dependent on the concentration. DHED was able to decrease the viral titers and suppress the entry, replication and assembly of the viral life cycles. Besides, the molecular experiment showed that ERK/MAPK pathway was involved in the anti‐PEDV mechanism of DHED [97].

Curcumin is a kind of polyphenols acquired from the rhizome of *Curcumae longae* L., the safety of curcumin has been approved by the US FDA [98]. Curcumin possesses a series of regulatory effects on biological function, including antioxidation, anti‐inflammation, and antitumor [99]. In regard to its antiviral effect, various viruses, such as HPV, HCV, and ZIKV, can be suppressed by curcumin according to previous reports [100]. In the virus‐induced porcine diarrhea, curcumin was proven effective in inhibiting PDCoV infection in LLC‐PK1 cells in a dose‐dependent manner. In addition, the application of curcumin mitigated the inflammation by inhibiting the expression of IRF3 and NF‐κB [68]. The antiviral capacity of curcumin on PEDV was also confirmed in a recent study. The in vivo model demonstrated that curcumin promoted the innate immune response of piglets through JAK‐STAT signaling pathway and thus inhibited the PEDV proliferation in the intestine [101]. Another study also uncovered the antiviral effect of curcumin on TGEV replication in a dose‐dependent manner. The application of curcumin at 40 μM significantly impeded the attachment of TGEV. Data also showed that curcumin also possessed the ability to directly eliminate TGEV, which was related to the concentration, temperature, and time of the curcumin application [102].

The flavonoid compounds showed broad antiviral potential according to recent studies. Myricetin is a kind of flavonoid extracted from the Cortex Myricae and many other plants [103]. The antiviral effect of myricetin against TGEV including its direct virucidal ability and inhibition of TGEV replication at a concentration of 100 mM. Besides, the myricetin inhibited CPE caused by TGEV infection in a dose‐dependent manner. The molecular docking analysis indicated the myricetin possesses the ability to competitively restricted the activity of TGEV PL^pro^ [71]. Another two flavonoids, chrysin, and naringenin, were screened from six molecules and showed antiviral capabilities at concentrations of 50 μg/mL (chrysin) and 25 μg/mL (naringenin) against PEDV infection by raising cellular survival rate and reducing viral titers. Besides, chrysin and naringenin targeted the viral post‐entry stage to restrict PEDV replication. The molecular docking indicated the interaction of chrysin and naringenin with PEDV 3CL^pro^ and PLP‐2, by which chrysin and naringenin blocked the function of these proteins and thus interfered with PEDV replication [72].

*Portulaca oleracea* L. (POL) is an herbal medicine broadly applied in the treatment of diabetes and diarrhea, the chemical component of POL and its pharmacology remains unclear. A recent study analyzed the constitution of polysaccharides in POL (POL‐P) and identified the antiviral effect of POL‐P on PoRV infection in suckling mice, evaluated by alleviated intestinal injury and reduced viral loads in jejunum and ileum. Further application of POL‐P in suckling piglets verified its protective effect, achieved by promoting IFN and IL‐10 levels in blood and recovered the balance of gut flora [73].

*Cryptoporus volvatus* is a special component in the TCM recipes, which was usually used for alleviating inflammation [104]. Ergosterol peroxide (EP) is a sterol compound and broadly detected in the mushroom *Cryptoporus volvatus*, *Poria cocos*, and *Cicada cordyceps* [105]. The mixture of EP and PDCoV showed the direct virucide effect of EP. Moreover, EP suppressed the PDCoV infection in a dose‐dependent manner and blocked the viral attachment and entry. The in vitro study also indicated that EP declined the function of NF‐κB and p38/MAPK pathways and decreased the production of cytokines to exert anti‐PDCoV abilities [106]. To further confirm the antiviral effect of EP, 7‐day‐old piglets challenged by PDCoV were adopted. The results showed that oral application of EP reduced the diarrhea ratio and mitigated the intestinal injury. Meanwhile, EP facilitated the elimination of PDCoV in intestine and reduced the virus‐induced cellular apoptosis [74]. Another study also indicated the anti‐PEDV ability of EP. EP significantly inhibited the whole life cycle of PEDV except the attachment. Mechanically, EP application restrained the PEDV‐induced apoptosis through interfering with the ROS production and p53 activation [75] (Figure 2).

Figure 2Chemical structures of main natural products effective in suppressing major SEVs infection. (A) Representative compounds from recipes effective in inhibiting PEDV and SADS‐CoV infection. (B) Representative compounds from single herb effective in controlling major SEVs infection. (C) Representative components from edible plants that inhibit major SEVs infection. (D) Representative natural metabolites from animals and plants that inhibit major SEVs infection. (E) Other natural products and derivatives identified for major SEVs control.

**Figure:**
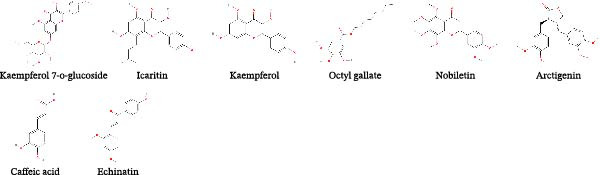
(A)

**Figure:**
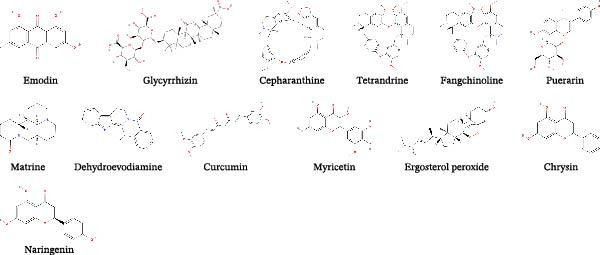
(B)

**Figure:**
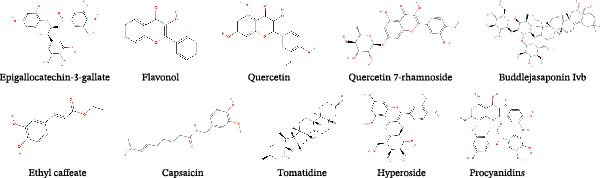
(C)

**Figure:**
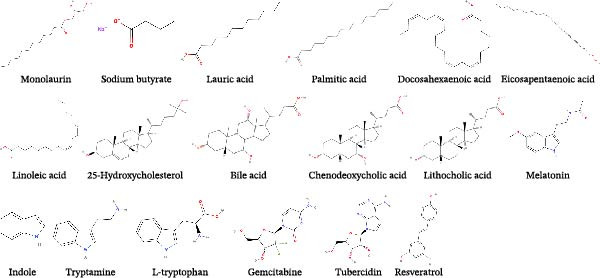
(D)

**Figure:**
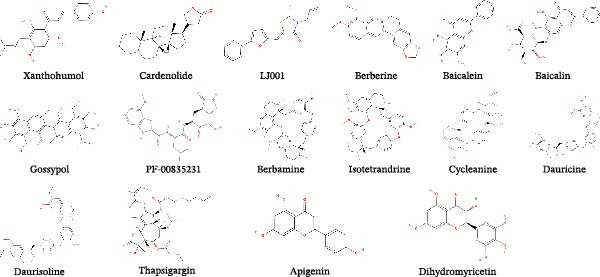
(E)

## 3. Compound From Edible Plants Effective in Controlling SEVs Infection

In addition to the medicinal herb, compounds from some edible plants also exhibited great potential in resisting viruses. Green tea is the most well‐known edible plant in China, which even derives tea culture in the normal life of Chinese. Epigallocatechin‐3‐gallate (EGCG) is the main phenol in green tea, which exerts antiviral effect on PEDV infection in vero cells in a concentration‐dependent manner (Table 2). Besides, EGCG was found to take effect at the attachment, entry, replication, and assembly stages of PEDV [107]. Flavonol, a flavonoid compound frequently extracted from tea and other herbs, exhibited anti‐PEDV abilities in vero and IPEC‐J2 cells with half antiviral concentration of 12.75 and 20.37 μM, respectively. The application of 80 μM flavonol inhibited PEDV replication and alleviated PEDV‐induced cytopathy in vero and IPEC‐J2 cells after PEDV infection at different MOI. Coincubation of flavonol and PEDV can maximize its antiviral capability, besides, flavonol mainly targeted the viral attachment and internalization. Of note, flavonol also exhibited antiviral effect against TGEV and PDCoV infection, which was probably related to its interaction with M^pro^ according to the molecular docking results [108].

**Table 2:** Effective compounds from edible plants for SEVs inhibition.

Compounds	Resource	Model	Targeting stages	Viruses	References
Epigallocatechin‐3‐gallate	Green tea	In vitro	Attachment, entry, and replication	PEDV	Huan et al. [107]
Flavonol	Tea	In vitro	Attachment and entry	PEDV, PDCoV, and TGEV	Liang et al. [108]
Quercetin	*Houttuynia cordata* Thunb.	In vitro and in vivo	—	PEDV	Gong et al. [109]
		In vitro and in vivo	Attachment and entry	SADS‐CoV	Feng et al. [110]
Quercetin 7‐rhamnoside	—	In vitro	Replication	PEDV	Choi et al. [111]
Griffithsin	Marine red algae	In vitro	Attachment and entry	PDCoV	Tang et al. [112]
Buddlejasaponin IVb	*Pleurotus ostreatus*	In vitro and in vivo	Replication and release	PEDV	Sun et al. [113]
Ethyl caffeate	*Bidens pilosa*	In vitro and in vivo	—	PEDV	Jiang et al. [69]
Capsaicin	Capsicum	In vitro	replication	TGEV	Zhang et al. [114]
Tomatidine	Tomatoes	In vitro	replication	PEDV and TGEV	Wang et al. [115]
Hyperoside	Hawthorn	In vitro and in vivo	—	PEDV	Wang et al. [116]
Procyanidins	Peanut	In vitro	Replication and release	PEDV	Liu et al. [50]

*Houttuynia cordata* Thunb. is a kind of edible vegetable popular in the southwest region of China, of which the quercetin (a flavonoid compound similar to flavonol) exerted anti‐PEDV effect in vero cells and in a piglet model [109]. The molecular docking results showed the binding of quercetin and PEDV 3CL^pro^, the fluorescence resonance energy transfer analysis also verified the inhibitory effect of quercetin on 3CL^pro^ [70]. Another study identified the intersecting targets of quercetin and diarrhea, and thus the antiviral capacity of quercetin against SADS‐CoV was verified. Quercetin at the concentrations of 25, 50, and 100 μM suppressed SADS‐CoV infection in a dose‐dependent manner in IPI‐FX cells. The whole lifecycles of SADS‐CoV were affected by quercetin, of which the attachment and entry stages were mainly targeted. Mechanically, the application of quercetin alleviated the accelerated cellular process induced by SADS‐CoV through regulating p53 transcription. The administration of quercetin in vivo further verified its antiviral potential on SADS‐CoV infection [110]. Furthermore, the hydroxylated product of quercetin, quercetin 7‐rhamnoside (Q7R) showed better antiviral ability with IC_50_ concentration of 0.014 g/mL, which is even better than ribavirin and interferon. Q7R inhibited PEDV infection mainly through impeding the replication period. Compared with PEDV, the antiviral effect of Q7R on TGEV is poor [111]. Red algae are a sort of green food with abundant protein and all of the essential amino acids for human, from which griffithsin (GRFT) is extracted belonging to lectin highly specific to mannose. A recent study investigated the antiviral effect of GRFT against PDCoV infection in IPI‐2I cells. The GRFT suppressed PDCoV‐infected ratio and viral titers at the concentration of 1 μg/mL and beyond. Besides, the time‐of‐addition analysis indicated its inhibitory effect on the viral attachment and entry stages. Mechanically, GRFT was able to wrap the virus by binding to PDCoV S protein and thus impede the viral entry [112]. Buddlejasaponin IVb (BJP‐IVb), a triterpenoid saponin extracted from *Pleurotus ostreatus*, has many bioactivities such as anti‐inflammation and antitumor [117]. In the past, it was applied to deal with ulcerative colitis and hyperlipidemia [118, 119]. Recently, the inhibitory effect of BJP‐IVb on PEDV infection was discovered, the replication and release periods were targeted by BJP‐IVb. Moreover, intramuscular injection of BJP‐IVb to the PEDV‐infected pigs alleviated clinical symptoms (such as diarrhea and vomiting) and promoted the intestinal mucosal repair [113]. Ethyl caffeate (EC) was identified by the binding ability to PEDV 3CL^pro^ from natural drugs database, the results showed that EC was effective in the PEDV‐challenged cell and pig models. The application of EC protected the piglets from death and decreased the viral load in the intestine [69]. Capsicum is well‐used in the food business to adjust the flavor of food, of which the capsaicin is the main ingredient to make it taste spicy and was applied to treat pain or inflammation. Of interest, capsaicin also exhibited antiviral effect on TGEV infection through inhibiting the replication stage. Meanwhile, the concentration of calcium ion participated in the antiviral mechanism, which was reduced by capsaicin via inhibiting the function of TRPV4 [114].

Of note, some researches in recent years revealed that some natural compounds derived from some fruits also exhibited antiviral abilities. Tomatidine, a steroidal alkaloid extracted from tomatoes, restricted the PEDV replication in vero and IPEC‐J2 cells. The combination of molecular docking, fluorescence spectroscopy, and isothermal titration calorimetry clarified the interaction of tomatidine and PEDV 3CL^pro^. The subsequent analysis by cleavage visualization and FRET affirmed the inhibitory effect of tomatidine on 3CL^pro^. The antiviral capacity against TGEV in vitro further showed the potential of tomatidine in treating viral infection [115]. Hyperoside is one of the main ingredients in hawthorn extracted by using ethanol method. The antiviral analysis showed the EC_50_ of hyperoside against PEDV is 2.588 μg/mL and the selection index is 9.72. Meanwhile, the administration of hyperoside prevented the infected piglets from death and significantly eliminate the viral load in intestine. The antiviral mechanism of hyperoside might be related to the interrupted interaction between PEDV N protein and p53 [116].

Procyanidins (PC) is a kind of pigment broadly existed in multiple plants, such as grape seeds, blueberries, apple peel, peanut, and whitethorn. PC belongs to polyphenols formed by the condensation of catechin and epicatechin and possesses powerful antioxidant property [120]. A recent study identified the anti‐PEDV ability of PC composed of catechin, procyanidin B1, procyanidin B2 and procyanidin A2. The PC inhibited PEDV infection in Vero E6 and IPEC‐J2 cells in a dose‐dependent manner, moreover, the PC restricted PEDV infection by targeting viral replication and release stages and direct viral inactivation effects. Inhibition of interferon production mediated by PEDV N protein was reversed by the application of the PC. The blockage of PEDV N protein by the PC also interfered with the viral binding to mitochondrial fusion protein 1 and alleviated the excessive mitochondrial fission, which was related to the infection of coronaviruses [50].

## 4. Restriction of Major SEVs by Natural Metabolites

### 4.1. Natural Metabolites From Animals

In recent years, increased attention on the antiviral roles of natural metabolites from animals and plants accelerated the development of antiviral candidate medicines. The most well‐investigated natural antiviral metabolites derived from animals could be the lipid substances. Monolaurin (ML) is broadly existed in the oil and breast milk, which is also produced from lauric acid (LA) [121]. High safety is the usual characteristic of natural metabolites. ML was listed in the generally recognized as safe (GRAS) compounds approved by the US FDA. In the piglet experiment, ML was intragastrically applied before PEDV infection, the usage concentration of 100 mg/kg body weight showed no effect on growth performance but obviously relieved the diarrhea (Table 3). Besides, the differential expressed genes between PEDV group and ML + PEDV group enriched in the interferon‐related pathways, which suggested the interferon‐regulated potential of ML and might help the recovery of intestine [122]. Recently, a study demonstrated the potential inhibitory effect of sodium butyrate (NaB), LA, palmitic acid (PA), docosahexaenoic acid (DHA), and eicosapentaenoic acid (EPA) on porcine coronaviruses including PEDV, TGEV and PDCoV. Among them, NaB showed anti‐PDCoV ability, LA showed anti‐TGEV ability and PA showed anti‐PEDV ability. However, DHA and EPA showed great potential in suppressing infection of PEDV, TGEV and PDCoV in Vero, PK‐15 and LLC‐PK1 cells, respectively. During PEDV infection, Endoplasmic reticulum stress (ERS) and inflammation of host were attenuated by DHA and EPA, accompanied by the enhancement of antioxidant level of host cells [123]. Some scientists had screened the intestinal contents of SPF‐pigs by using proteomics and metabolomics and acquired 10 metabolites. Among these metabolites, linoleic acid showed great capacity in inhibiting PEDV replication and release, the in vivo experiment also verified the protective ability of linoleic acid at 0.05 g/meal per pig against PEDV infection in piglets [124]. The manipulation of fatty acids showed the potential of developing lipids as antiviral candidate to control SEVs transmission.

**Table 3:** Effective natural metabolites from animals and plants for SEVs inhibition.

Metabolites	Derivative source	Model	Targeting stages	Viruses	References
Monolaurin	Lauric acid	In vivo	—	PEDV	Zhang et al. [122]
Butyrate NaB	Fatty acids	In vitro	—	PDCoV	Suo et al. [123]
Lauric acid	Fatty acids			TGEV	
Palmitic acid	Fatty acids			PEDV	
Docosahexaenoic acid	Fatty acids			PEDV, PDCoV, and TGEV	
Eicosapentaenoic acid	Fatty acids				
Linoleic acid	Fatty acids	In vitro and in vivo	Replication and release	PEDV	Yang et al. [124]
Long‐chain ceramides	Ceramides	In vitro	Replication	PoRV	Tao et al. [125]
25‐Hydroxycholesterol	Cholesterol	In vitro and in vivo	Replication	PDCoV	Ke et al. [126] Zhang et al. [122] Zhang et al. [114]
		In vitro	Entry	SADS‐CoV	Liu et al. [127]
Histone deacetylase 6	Enzyme	In vitro	Replication	PDCoV	Li et al. [128]
Monoglycerides	Fatty acids	In vivo	—	PEDV	Phillips et al. [129]
Chenodeoxycholic acid	Bile acids	In vitro	Replication	PDCoV	Kong et al. [130]
Lithocholic acid					
Melatonin	Indoles	In vitro	Entry and replication	PEDV, PDCoV, and TGEV	Zhai et al. [131]
Xanthohumol	—	In vitro	—	PEDV	Lin et al. [132]
Cardenolide	Steroids	In vitro	—	TGEV	Yang et al. [133]
Resveratrol	Polyphenols	In vitro	Replication	PDCoV	Wang et al. [134]
Gemcitabine	Nucleoside analog	In vitro	—	PEDV	Zheng et al. [135]
Tubercidin	Nucleoside analog	In vitro	Replication	PEDV and SADS‐CoV	Wang et al. [116]
CATH‐B1	Chicken antimicrobial peptides	In vitro	—	PEDV	Pashaie et al. [136]
LL‐37	Human antimicrobial peptides		—		
APB‐13	Bovine antimicrobial peptides	In vitro and in vivo	Replication	TGEV	Liang et al. [137]
PABPC4	Poly(A)‐binding protein	In vitro	—	SADS‐CoV	Jiao et al. [138]

The untargeted liquid chromatography mass spectrometry (LC‐MS) analysis pointed out that 451 lipids were significantly changed after the PoRV infection in IPEC‐J2 cells. Notably, all of the ceramides, especially long‐chain ceramides, levels were increased by the PoRV infection. The addition of long‐chain ceramides significantly inhibited PoRV infection and mainly targeted the viral replication stage. Accordingly, the application of ceramides inhibitors promoted PoRV replication, and activation of long‐chain ceramides production significantly inhibited PoRV infection and showed broadly antiviral effects on PoRV genotypes and bovine RV strains. The anti‐PoRV ability of ceramides was related to the induced apoptosis, which could be reversed by the application of apoptosis inhibitor [125].

25‐Hydroxycholesterol (25HC) is a kind of oxysterol derived from cholesterol, which involved the catalysis of cholesterol‐25‐hydroxylase (CH25H). As a secondary metabolite of cholesterol, 25HC functions as inhibitor of cholesterol in the negative feedback system of cholesterol biosynthesis [139]. 25HC were proven reliable in resisting various viruses such as HIV, ZIKV, and DENV [65]. The antiviral capacity of CH25H was first discovered, thus inspired the exploration of 25HC. PDCoV infection stimulated the expression of CH25H in IPI‐FX cells and the overexpression of CH25H inhibited the replication of PDCoV [126]. The subsequent study focused on the direct antiviral effect of 25HC on PDCoV infection. 25HC significantly suppressed the post‐entry stage of PDCoV, the time‐of‐addition assay showed that transitory addition of 25HC during the early and middle period of post‐entry stage of PDCoV was effective in viral resistance [140]. Mechanically, the infection of PDCoV involved the manipulation of host cholesterol metabolism, which was recovered by the administration of 25HC. 25HC declined the free cholesterol accumulation induced by PDCoV and promoted its esterification and storage in lipid droplets, in which the autophagy flux and lysosomal function plays crucial roles. The inhibition of TFEB‐mediated lipophagy attributed to the anti‐PDCoV effect of 25HC. After that, the administration of 25HC in piglets model further affirmed the anti‐PDCoV potential of 25HC in the swine industry [141]. Liu et al. [127] found that 25HC suppressed SADS‐CoV infection in IPI‐2I and Vero E6 cells in a dose‐dependent manner. Furthermore, 25HC targeted viral entry rather than attachment and release to impede the SADS‐CoV infection. The blockade of S protein‐mediated membrane fusion contributed to the antiviral effect of 25HC, verified in Vero E6 and HEK293T cells by immunofluorescent analysis.

Similar to CH25H, another host enzyme, histone deacetylase 6 (HDAC6) was shown to participate in the resistance of viral replication. HDAC6 is not only a deacetylase but also with ubiquitin E3 ligase activity. PDCoV infection caused reduction of HDAC6 level in dose‐dependent and time‐dependent manners. The overexpression of HDAC6 significantly suppressed the acetylation of α‐tubulin and PDCoV replication, on the contrary, the application of tubacin (a HDAC6 inhibitor) and HDAC6‐specific siRNA both increased the acetylation of α‐tubulin and accelerate the infection of PDCoV in a dose‐dependent manner. Mechanically, HDAC6 showed interaction with nsp5 and nsp8 of PDCoV, and the ubiquitination of nsp8 by HDAC6 was based on its deacetylase function. The mutation of K46 and K58 of nsp8 could antagonize the degradation effect of HDAC6 and counteract its anti‐PDCoV ability [128]. These data suggested the antiviral effect of HDAC6 on PDCoV, however, the relation between HDAC6 and porcine coronaviruses seems complex. Another study further investigated the antagonism of PDCoV on HDAC6, the PDCoV nsp5 cleaved the HDAC6 at Q519 site to two segments (amino acids 1–519 and 520–1159), after the cleavage, HDAC6 lost the ability to degrade PDCoV nsp8 and the ability to induce IFN response by RIG‐I activation. In addition to PDCoV, TGEV, PEDV, and SADS‐CoV also exhibited similar mechanisms to resist the HDAC6‐mediated antiviral response of the host [67]. Multiple viruses including SEVs could survive in the feed to induce porcine infection and economic loss. Medium chain fatty acids (MCFAs) are effective in inhibiting viruses by forming micelles [142]. Interestingly, monoglycerides also possess the ability to form micelles, which requires lower concentration than MCFAs [143]. These results indicated the antiviral potential of monoglycerides. During a feed‐contaminated model, piglets were challenged by PEDV for 20 days. At the same time, experimental monoglyceride blend (EMB) was administrated with the feed. Notably, at the end of this study, 54.8% PEDV‐positive piglets were detected in the untreated group. However, all of the EMB application at concentrations of 1.5, 2.5 and 3.5 kg/t feed showed 100% protective effects against PEDV infection [129].

Some metabolites were shown as double‐edged sword in viral infection. In a piglet model challenged by SADS‐CoV, the untargeted metabolomic analysis indicated the significant change of bile acid (BA) in the infected piglets. Most of the BAs were reabsorbed in the ileum, which in turn results in the highest concentration of BA in ileum [144]. Meanwhile, some of the SEVs were shown to target ileum for rapid infection and replication. The relation between BA and SEVs infection in ileum was unclear. The researchers constructed stem cell‐derived porcine intestinal enteroid and verified that specific BA such as cholic acid promoted the SADS‐CoV replication in the early stage. Besides, the BA induced the caveolae‐mediated endocytosis and the change of endosome/lysosome including the pH decrease of endosome, which facilitated the replication of SADS‐CoV [145]. However, another study regarding BA suggested the antiviral effects of chenodeoxycholic acid (CDCA) and lithocholic acid (LCA) against PDCoV. The data showed that CDCA and LCA at the concentration of 100 and 12.5 μM, respectively, suppressed the viral titers of PDCoV in the LLC‐PK1 cells and IPEC‐J2 cells. Furthermore, the production of IFN‐λ3 and ISG15 were induced through G protein‐coupled receptor by CDCA and LCA, which resulted in the inhibition of PDCoV replication during the post‐entry stage [130].

Indoles and derivatives were also discovered antiviral abilities against SEVs infection. Melatonin, a compound belongs to indoles, is broadly exists in the plants, animals and fungi. In the animal organisms, melatonin is distributed in multiple organs and tissues [146]. Melatonin was able to alleviate the pathological injury caused by EBOV and VEEV infection [147, 148]. Recently, a study systematically evaluated the antiviral effects of indoles on porcine coronaviruses. Melatonin, indole, tryptamine, and L‐tryptophan exhibited inhibitory abilities against PDCoV, PEDV, and TGEV infection. Moreover, the viral entry and replication stages were targeted by melatonin for blockage of PDCoV, TGEV, and PEDV infection in LLC‐PK1, PK15, and vero cells, respectively [131].

### 4.2. Natural Metabolites From Plants

Natural metabolites derived from plants also showed antiviral potential in SEVs control. Xanthohumol is extracted from *Humulus lupulus*, which has a series of pharmacological activities, including anticancer and antivirus. Xanthohumol was shown to target M^pro^ of coronavirus for inhibition of SARS‐CoV‐2 and PEDV, of which the IC_50_ was 1.53 and 7.51 μM, respectively [132]. Cardiac glycosides, also known as cardenolides, are special steroidal metabolites produced in various plant families, such as *Nerium oleander*, *Prunus persica*, *Plantago asiatica*, and Brassicaceae, serving a protective role in plants [149]. Cardenolides were utilized for the clinical treatment of cardiovascular diseases because of its inhibition of Na^+^−K^+^ pump. Cardenolides suppressed viral titers of TGEV in a dose‐dependent manner with a IC_50_ as 37 nM and inhibited the production of IL‐6 [150]. The Na^+^−K^+^‐ATPase was involved in the anti‐TGEV mechanism. Besides, a JAK1‐mediated proteolysis also participated in the anti‐TGEV effect of cardenolides, which was Na^+^−K^+^‐ATPase independent [133] (Figure 3). Resveratrol (Res), a polyphenolic compound, is a kind of self‐protective factor produced by various plants during stress or stimulation. The application of 50 μM Res significantly suppressed the viral titers and expression of PDCoV N, besides, the time‐of‐addition analysis showed that Res mainly targeted the viral replication stage. Mechanically, the application of Res inhibited the PDCoV infection by activating SIRT1 and phosphorylation of IRF3 and enhancing the production of IFN‐β [134].

Figure 3Schematic diagram of anti‐SEVs natural products and representative infectious process displayed using coronavirus. (A) Transmission route of major SEVs and representative natural products effective in treating virus in feed and intestines of piglets. (B) Representative infection cycle of virus, including attachment, replication, maturation, assembly, and release. Representative natural products that possess direct virucidal ability and target viral life cycles. The picture was drew by using BioGDP [151].

**Figure:**
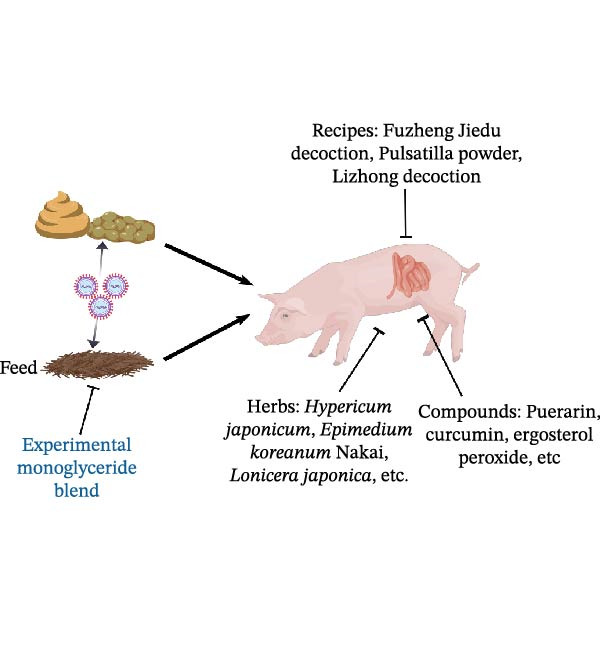
(A)

**Figure:**
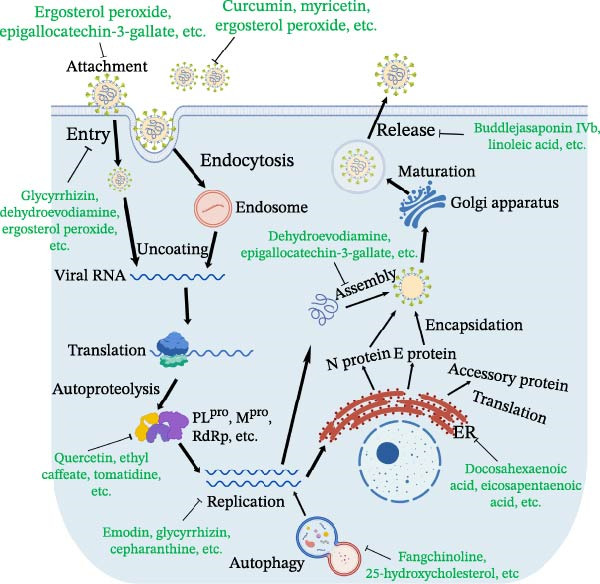
(B)

### 4.3. Key Substances in Organisms

Recent studies have found that some key substances in organisms, such as nucleoside and peptide, show antiviral capabilities. A nucleoside analog, names as gemcitabine, has similar structure with cytidine and deoxycytidylate. In an in vitro model, gemcitabine suppressed the PEDV infection and decreased the viral titers with a EC_50_ of 3.12 μM. Considering that baicalein has direct virucidal effect, the combination of gemcitabine (1 μM) and baicalein (1.5 μM) significantly inhibited the infection of PEDV and showed better antiviral efficacy [135]. Another study had screened 206 natural products and focused on the nucleoside analog obtained from *Streptomyces tubercidicus*, which named as tubercidin. Data demonstrated that tubercidin at 1 μM exhibited great antiviral ability to PEDV infection inVero and LLC‐PK1 cells and mainly targeted the post‐entry stage. The molecular docking result suggested the binding of tubercidin with RNA‐dependent RNA polymerase (RdRp), which implied its broad antiviral capabilities. Furthermore, the application of tubercidin also significantly suppressed the infection of SADS‐CoV and PRRSV in a dose‐dependent manner [152].

Antimicrobial peptides (AMPs) from different species exhibited broad antibacterial abilities and antiviral effects on multiple viruses. In a vero cell model, AMPs from chicken (CATH‐B1) and human (LL‐37) showed dramatically inhibitory effect on PEDV infection at concentrations of 5 and 10 μM, whereas four AMPs derived from swine (PMAP‐36, PMAP‐23, PR‐39, and PG‐1) displayed no suppressing ability. The pretreated CATH‐B1 and LL‐37 and coincubation with PEDV are both effective in reducing PEDV replication. Besides, the transmission electron microscopy results demonstrated that both CATH‐B1 and LL‐37 were able to change viral morphology and induced abnormal aggregation of virions [136]. With regard to TGEV infection, researchers have identified a bovine AMP (APB‐13) effective for the viral inhibition. Application of APB‐13 (concentration of 0–62.5 μg/mL) exerted antiviral effects in a dose‐dependent manner and mainly affected the replication stage of TGEV. In the piglet model, the administration of APB‐13 significantly repaired the intestinal villi destroyed by TGEV and decreased viral shedding [137]. These results indicate the antiviral potential of peptides from organisms and probable mechanism that the interaction between peptides and virus negatively affects viral infectivity.

PABPC4 belongs to a sort of poly(A)‐binding protein, which participates in the regulation of stress response and mRNA homeostasis. A recent study found that SADS‐CoV infection inhibited the PABPC4 expression in a time‐dependent manner, while the overexpression of PABPC4 showed inhibitory effect on SADS‐CoV replication. Further investigation indicated that PABPC4 degraded SADS‐CoV N protein by PABPC4/MARCHF8/NDP52 mediated autophagy [153].

## 5. Discussion and Perspectives

In the past years, hundreds of antiviral medicines were discovered for the SEVs control and diarrhea treatment. Except for the natural products, various small chemical molecules were also applied into the inhibition of SEVs. For example, a rhodanine derivative LJ001 suppressed the replication of TGEV and PDCoV in ST cells with a concentration of 12.5 μM [154]. However, natural products are characterized with low toxicity and high production, which is critical for the drug investigation and brings great advantages to the development of natural products.

From the TCM perspective, the key point to cure infectious disease is to enhance immunity and recover the homeostasis and balance of host. Therefore, the application of multiple compounds from natural products listed in this manuscript showed ability to regulate immune response by upregulating IFN response and relieving inflammation. Moreover, in consideration of the manipulation of metabolism process of host by viruses, extra supplementation of metabolites also helped to suppress abnormal accumulation and restore the host metabolic balance. Among the candidate antiviral drugs reported in the current study, most of the compounds still stay at the laboratory development period, their potential effects on viral infection in pigs are far from verified. There are two major reasons: i) most of the compounds are insoluble in the water, the delivery strategies remain unclear, ii) most of the natural products were investigated via in vitro study, their pharmacokinetics and bioavailability are unknown in pigs. In detail, the solubility affects the final dosage form of products, besides, the effective concentration of active compounds varies in different solvent. To solve this problem, the compounds can be enveloped by delivery vehicles, such as HβCD, for high water solubility. In addition, a study integrated the antiviral candidate glycyrrhizic acid and nanoparticles to acquire a novel product GANPs with high biocompatibility. The application of GANPs showed antiviral effects on MHV‐A59 and SARS‐CoV‐2, moreover, the high targeting ability of GANPs in animal model indicated its developmental potential [155]. A chitosan‐coated nano‐liposome vehicle used for berberine delivery showed great stability and delayed release in the intestine, which further increased the bioavailability [156]. Fermentation of TCM also facilitates the development of antiviral drugs, including increasing the yield of activities and enhance therapeutic effects [157]. Treated Pulsatilla powder after fermentation exerted better therapeutic effect on piglets challenged by PEDV than application of direct extract [51]. The cross‐discipline studies bring great inspiration to the anti‐SEVs drug development in the future. The in vitro studies using cell models benefits from the advantages of these approaches, such as simple protocols, simple instruments, short experiment time, and easy to reproduce. However, SEVs are far from susceptible in the enteric cell lines in vitro, most of the researches have to be conducted in the renal cell lines. This phenomenon makes the conclusion of in vitro study unable to guide the clinical medication in the pig industry. The culture of organoid that mimics the tissue construction and microenvironment of intestine is urgent for the development of anti‐SEVs drugs. In recent years, a novel porcine intestinal enteroids were established using crypts of duodenum, jejunum and ileum, which is applicable as SEVs infection model [158].

Multiple natural products were screened by chemical database from FDA, whereas the evaluation process consists of PCR reaction and viral titers measurement, which needs plenty of time and repeated work. Nowadays, with the development of artificial intelligence (AI), the discovery of medicine become faster and batch processing [159]. According to the broad antiviral target, such as 3CL^pro^, M^pro^, and RdRp, researchers utilized AI to recognize two flavonoids baicalein and baicalin effective for PEDV inhibition [160], xanthohumol effective for SARS‐CoV‐2 and PEDV inhibition [132], gossypol effective for SARS‐CoV‐2 and PDCoV inhibition [161]. An inhibitor of 3CL^pro^ (PF‐00835231) screened from FDA‐approved compounds showed anti‐TGEV ability by targeting the cleavage site of nsp5–nsp6 and inhibiting the enzyme activity of nsp5. Meanwhile, PF‐00835231 also suppressed SADS‐CoV infection in mice and decreased the viral copies [162]. Nirmatrelvir, an inhibitor of SARS‐CoV‐2 3CL^pro^, showed limited antiviral effect on PDCoV infection. However, after the modification of nirmatrelvir based on the characteristic of PDCoV S2 pocket, the novel derivative T1 showed enhanced antiviral effect on PDCoV infection in vivo and in vitro [163]. In addition, based on the antiviral ability of CEP, after the biosynthetic gene mining and metabolomics analysis, seven analogs (including TED, berbamine, Fan, isotetrandrine, cycleanine, dauricine, and daurisoline) were identified with broad anti‐coronavirus abilities, including SARS‐CoV‐2, SADS‐CoV, and PEDV [64]. This study verified the application potential of structure–activity theory in identifying antiviral medicines and promotes the discovery of medicinal plants. From another perspective, the online databases concerning TCM pharmacology and genes, such as TCMSP, GeneCards, UniProt and SwissDrugDesign databases, were used to identify the potential anti‐PEDV target of berberine, further recognition was conducted by combining enrichment analysis and molecular docking [164], this research mode facilitates the excavation of target of viral infection and in turn benefits the development of antiviral drugs.

With regard to the discovery of novel anti‐SEV drugs, targeting key proteins related to viral replication and invasion also provide new strategies. ERS was shown related to multiple viral infection, an inducer of ERS (thapsigargin, TG) exhibited anti‐TGEV effects in IPEC‐J2 cells and an intestinal organoid model, besides, oral application of TG also suppressed TGEV infection in piglets and alleviated intestinal injury caused by TGEV [165]. In addition to ERS, the integrated stress response (ISR) also participated in the antiviral response of host. A recent study identified a sort of new compounds inducing ISR by using molecular design strategy and synthesized four lead compounds. Lead compounds 1‐B and 1‐C inhibited PEDV and PDCoV infection in a dose‐dependent manner in vero and LLC‐PK1 cells, mechanically, phosphorylation of eIF2α was upregulated to block the protein synthesis of host, which resulted in the inhibition of PEDV and PDCoV replication [166].

The utilization of CRISPR/Cas9 system accelerates the discovery of host protein YIPF5 important for PEDV infection [167], and the combination of CRISPR/Cas9 and AI regarding critical proteins like YIPF5 brings exciting prospects to the investigation of antiviral medicine. In addition, the CRISPR/Cas9 system facilitates the establishment of engineered virus platform for the screening of antiviral drugs. Fang et al. [168] constructed a novel PDCoV expressing nanoluciferase by replacing the gene NS6. Based on this virus, the team identified the anti‐PDCoV ability of lycorine (an alkaloid extracted from *Lycoris radiata Herb*.) in a dose‐dependent manner using LLC‐PK1 cell model. Whereas the Res screened in this study showed no antiviral effect on PDCoV in LLC‐PK1 cells, which contradicted the result in the reference 146, indicating the antiviral effect of Res was cell‐line‐dependent. Zhang et al. [169] constructed a PDCoV reporter virus by introducing the enhanced green fluorescent protein based on the TAR cloning technology in yeast. Then Zhang et al. [169] identified three compounds (CGA, apigenin, and dihydromyricetin) from the medicinal food homology compounds library, of which the selective index against the reporter virus was 57.49, 58.71, and 26.38, respectively. The antiviral effects of these compounds were further verified in LLC‐PK1 and ST cells by measuring the viral titers, genome copies and expression of PDCoV N.

Therefore, the natural product is a useful library for antiviral candidate molecules, besides, novel technologies and target‐directed development strategy also offer a new reliable paradigm to accelerate the discovery of antiviral drugs.

## 6. Conclusion

In conclusion, SEVs are closely associated with both animal welfare and human health, positioning them as important issue restricting the economic development in pig industry. Outbreaks and consistent prevalence can cause coinfections and further trigger novel variants with higher pathogenicity. Therefore, natural products with medicinal potential are worthwhile for more attention in developing medicines against SEVs, on account of their multiple antiinfection capacities and low costs, we will be better prepared to handle sudden infectious diseases in the future by drawing on the experience we are gaining now.

## Funding

This work was supported by the National Natural Science Foundation of China (Grants 32503119 and 32473027), the Fundamental Research Funds for the Central Universities (Grants SWU‐KQ25021 and SWU‐XJPY202305), the Natural Science Foundation of Chongqing, China (Grant CSTB2025NSCQ‐GPX0489), the Chongqing Modern Agricultural Industry Technology System (Grant CQMAITS202512), and the National Center of Technology Innovation for Pigs (Grant NCTIP‐XD/C17).

## Disclosure

Due to the space limitations, the authors apologize that some excellent works were not cited in this review.

## Conflicts of Interest

The authors declare no conflicts of interest.

## Data Availability

Data sharing is not applicable to this article as no datasets were generated or analyzed during the current study.
